# Minocycline modulates microglia polarization in ischemia-reperfusion model of retinal degeneration and induces neuroprotection

**DOI:** 10.1038/s41598-017-14450-5

**Published:** 2017-10-25

**Authors:** Amel Ahmed, Lei-Lei Wang, Safaa Abdelmaksoud, Amal Aboelgheit, Safaa Saeed, Chun-Li Zhang

**Affiliations:** 1 Department of Molecular Biology, University of Texas Southwestern Medical Center, Dallas, Texas 75390 USA; 2 Hamon Center for Regenerative Science and Medicine, University of Texas Southwestern Medical Center, Dallas, Texas 75390 USA; 3 Department of Histology and Cell Biology, Faculty of Medicine, Assiut University, Assiut, Egypt

## Abstract

Retinal ischemia-reperfusion (IR) injury causes irreversible loss of neurons and ultimately leads to permanent visual impairment and blindness. The cellular response under this pathological retinal condition is less clear. Using genetically modified mice, we systematically examined the behavior of microglia/macrophages after injury. We show that IR leads to activation of microglia/macrophages indicated by migration and proliferation of resident microglia and recruitment of circulating monocytes. IR-induced microglia/macrophages associate with apoptotic retinal neurons. Very interestingly, neuron loss can be mitigated by minocycline treatment. Minocycline induces *Il4* expression and M2 polarization of microglia/macrophages. IL4 neutralization dampens minocycline-induced M2 polarization and neuroprotection. Given a well-established safety profile as an antibiotic, our results provide a rationale for using minocycline as a therapeutic agent for treating ischemic retinal degeneration.

## Introduction

Retinal ischemia is a common cause of irreversible blindness^1^ in a wide range of retinal degenerative diseases such as glaucoma, diabetic retinopathy, retinal artery occlusions, and retinopathy of prematurity^2–6^. An experimental ischemia-reperfusion (IR) model can be created by raising the intraocular pressure above systolic pressure (to induce acute retinal ischemia with ensuing deprivation of oxygen and nutrients), followed by releasing the pressure to cause a subsequent reperfusion injury, which exacerbates neuron degeneration by glutamate excitotoxicity^5,7,8^and production of reactive oxygen species^5,9–11^. Since retinal neurons lack the ability to regenerate after injury, neuron loss in IR is irreversible and ultimately leads to permanent visual impairment or blindness^12^. The current neuroprotective therapeutics that directly targets the cell death cascade for retinal degeneration is generally ineffective^13,14^. Therefore, we explored an alternative strategy by modulating the pathological environment with a clinically relevant small molecule.

In response to neural damage, local microglia become activated with changes in morphology, gene expression, proliferation, and migration^15–17^. Microglia activation can worsen retinal diseases by releasing a wide range of toxic and pro-inflammatory mediators^16^. Activated microglia phagocytose not only the dead and dying retinal neurons but also the damaged living ones that otherwise may be able to survive^18,19^. Circulating immune cells such as macrophages also contribute to the inflammatory response surrounding the degenerative retinal area^16^. These recruited macrophages help achieve tasks that cannot be efficiently accomplished by local microglia^20^.

Interestingly, microglia and macrophages can functionally switch between the M1 and M2 phagocyte types, depending on the local environment, a process termed polarization^21^. M1 phagocytes are involved in neuronal degeneration and dysfunction of the neural network through their production of several pro-inflammatory cytokines and mediators^21^. In contrast, M2 phagocytes inhibit inflammation and promote tissue remodeling through altered gene expression (such as ARG1, YM1 and CD206) and the production of neuroprotective factors (such as BDNF, GDNF, TGF, and IGF1)^21–23^. A shift of M2 into M1 phagocytes was shown to play a deleterious role in many neurological disorders^24^. As such, enhancing the neuroprotective effect of myeloid cell polarization to M2 starts to emerge as a potential therapeutic strategy. To date, however, microglia/macrophage polarization has not been well examined in the IR model of retinal degeneration.

In this study, we examined whether degenerative retinopathy can be mitigated by targeting the pathological microenvironment, which consists of activated microglia/macrophages. We first extensively analyzed the behavior of microglia/macrophages in response to retinal IR injury. We then studied the effect of minocycline, a clinically available tetracycline antibiotic, on the pathological environment and neurodegeneration. Besides its known anti-microbial activity, minocycline can cross the blood retinal barrier and exert anti-inflammatory, anti-apoptotic and neuroprotective effects. The protective effects of minocycline have been attributed to its ability to modulate microglia activation and polarization^25–34^. Our results revealed that activated microglia/macrophages contribute to neuron loss in the retinal IR model and that this loss can be significantly mitigated by minocycline treatment post injury.

## Results

### IR injury alters microglia morphology and distribution

**Figure 1:**
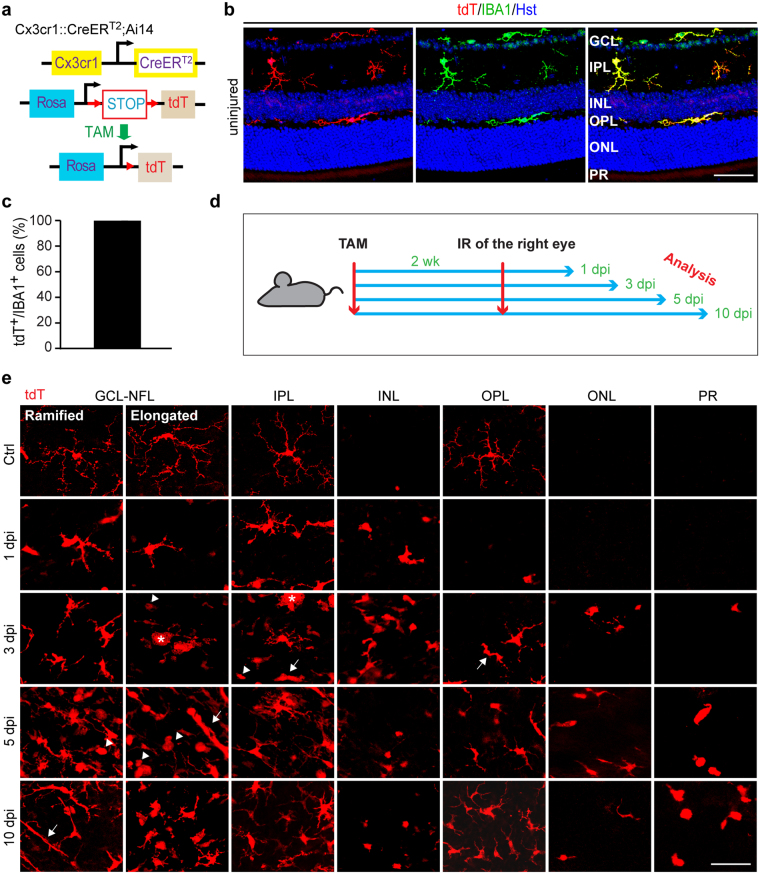
IR induces robust activation of retinal microglia. (**a**) A genetic approach to trace retinal microglia in transgenic *Cx3cr1::CreER* ^*T2*^ *;Ai14* mice (TAM, tamoxifen; tdT, tdTomato). (**b**) Representative confocal images of retinal microglia (GCL, ganglion cell layer; IPL, inner plexiform layer; INL, inner nuclear layer; OPL, outer plexiform layer; ONL, outer nuclear layer; PR, photoreceptor layer). Scale bar, 50 µm. (**c**) Labeling efficiency of resident microglia by the reporter tdT (n = 20 mice). (**d**) A schematic diagram of experimental design (IR, ischemia reperfusion; wk, weeks; dpi, days post injury). (**e**) Representative confocal images of microglia in retinal whole mounts. Resting microglia exhibit ramified morphology with small soma and long thin primary processes. At the indicated time points, injury induces soma enlargement and process retraction. The arrowheads, arrows, and asterisks indicate amoeboid, rod-like, and giant cells, respectively. NFL, nerve fiber layer; scale bar, 50 µm.

IR injury-induced microglia plasticity was examined by their morphology and distribution along the retinal layers at different time points post injury. In addition to immunohistochemistry, microglia were also genetically labeled in the *Cx3cr1::CreER* ^*T2*^ *;Ai14* reporter mice, in which the *CreER* ^*T2*^ gene was knocked into the endogenous *Cx3cr1* locus^35^ and the *tdTomato* (tdT) reporter gene under the control of the *CAG* promoter was knocked into the *ROSA* locus^36^. When these mice were treated with tamoxifen (TAM) for 4 days and examined 2 weeks later, ramified microglia morphology^37^ could be clearly observed by the microglia-specific expression of tdT (Fig. 1a,b). Quantification showed that about 99% IBA1^+^ cells (n = 20 mice) in the retina were tdT^+^ (Fig. 1b,c), indicating that a vast majority of endogenous microglia can be genetically labeled under our experimental condition.

Two weeks after TAM treatment of the reporter mice, IR injury was performed on the right eye while the uninjured left eye served as a control. Mice were then sacrificed at 1, 3, 5 and 10 days post injury (dpi) (Fig. 1d). Both retinal cryosections and flat mounts were used to better understand cell morphology and distributions among different retinal layers.

In retinal flat mounts of control eyes (Fig. 1e), tdT^+^ cells showed a general branched morphology with small soma from which long primary processes extended radially and gave rise to shorter secondary ones. Two morphological types were distinguishable in the ganglion cell layer (GCL) and nerve fiber layer (NFL): ramified cells and elongated cells. The ramified cells have triangular or ovoid somas and long thin-branched primary processes. Those with elongated morphology have ellipsoid-shaped somas, with their long primary processes arising mainly from the edges. tdT^+^ cells in the inner plexiform layer (IPL) exhibited a less ramified morphology. The divergence of the primary processes of these cells was limited only to the periphery, which is unlike the primary processes of ramified cells in the GCL-NFL, whose bifurcation into secondary processes occurred along their whole length. In the outer plexiform layer (OPL), ramified tdT^+^ cells differed from their counterparts in the GCL-NFL and IPL by having many short thin processes arising from both the primary and secondary processes. When injured eyes were compared to control ones (Fig. 1e), tdT^+^ cells showed soma enlargement with shortened and thickened processes. At 3 dpi, two new morphological types started to appear in the injured eye, with increased abundance at 5 dpi. These 2 types are: amoeboid cells, with rounded somas and no processes, and rod-like cells, with stretched somas and two processes arising from each pole of the soma. In some instances, the amoeboid cells acquired large somas and multiple nuclei, the shape of cells known as multinucleated giant cells.

**Figure 2:**
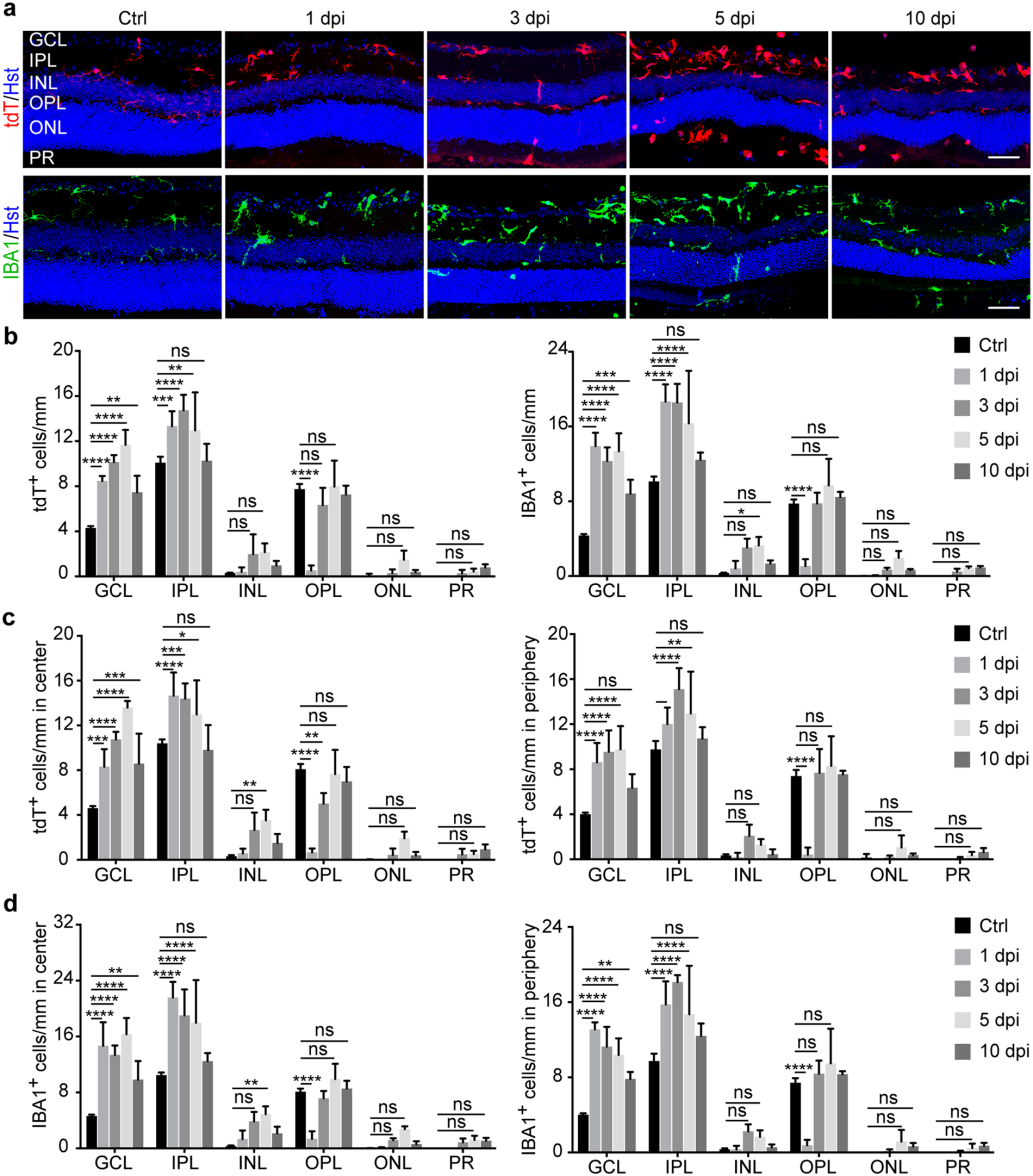
IR alters microglia orientation and distribution. (**a**) Confocal images of retinal sections showing orientation and layer distribution of microglia under resting and IR injury conditions. Microglia were mainly observed in the GCL and IPL but rarely in the OPL at 1 dpi. They gradually appeared in the INL, ONL, and PR at later time points after injury. Scale bars, 50 µm. (**b**) Retinal layer distribution of microglia under the indicated conditions. Data are presented as mean ± SD per mm retinal length (n = 4 mice per group). Statistical analysis was performed by two-way ANOVA and post hoc Tukey’s test (*p ≤ 0.05, **p ≤ 0.01, ***p = 0.0004, and ****p ≤ 0.0001; ns, not significant). (**c**) Subregional analysis of tdT^+^ cells under the indicated conditions. Data are presented as mean ± SD per mm retinal length (n = 4 mice per group). Statistical analysis was performed by two-way ANOVA and post hoc Tukey’s test (*p = 0.03, **p ≤ 0.01, ***p = 0.0002, and ****p ≤ 0.0001; ns, not significant). (**d**) Subregional analysis of IBA1^+^ cells under the indicated conditions. Data are presented as mean ± SD per mm retinal length (n = 4 mice per group). Statistical analysis was performed by two-way ANOVA and post hoc Tukey’s test (**p ≤ 0.01 and ****p ≤ 0.0001; ns, not significant).

When the orientation of tdT^+^ and IBA1^+^ cells was examined through the layers in retinal sections (Fig. 2a), the processes of the GCL ramified cells in control eyes were penetrating the layer perpendicularly, directed up and/or down, while those of elongated cells were running parallel to the retinal surface. Those in the IPL of control eyes were distributed within the layer in 3 areas: either close to the GCL or INL or in the middle perpendicular to the retinal surface. The main processes of those next to GCL or INL, or they were running parallel to these layers, while their end processes sometimes reached into the GCL or INL. In some instances, the processes of tdT^+^ and IBA1^+^ cells in the OPL of control eyes extended to the INL and ONL. Quantification showed that tdT^+^ and IBA1^+^ cells were mainly seen in the GCL, IPL and OPL (Fig. 2a,b), and they were evenly distributed throughout the retina from the center to the periphery (Fig. 2c,d).

IR injury completely disrupted the above stereotypical orientation and distribution of tdT^+^ and IBA1^+^ cells (Fig. 2a,b). Their distribution along the retinal layers noticeably changed in the center and the periphery with many cells in the INL, ONL and photoreceptor (PR) layers (Fig. 2a–d). At 1 dpi, they appeared mainly in the GCL and IPL and were rarely seen in the OPL (Fig. 2a,b). Afterwards, their presence in the OPL increased at 3 dpi, with some cells starting to appear in the INL, ONL and PR layers. The IBA1^+^ cells increased at 5 dpi but then decreased at 10 dpi with the exception of those in the PR layer (Fig. 2a,b). Together, these observations indicate that IR injury induces robust microglial activation that is manifested by their morphological changes and altered distribution among retinal layers.

### IR injury induces microglia proliferation and macrophage recruitment

**Figure 3:**
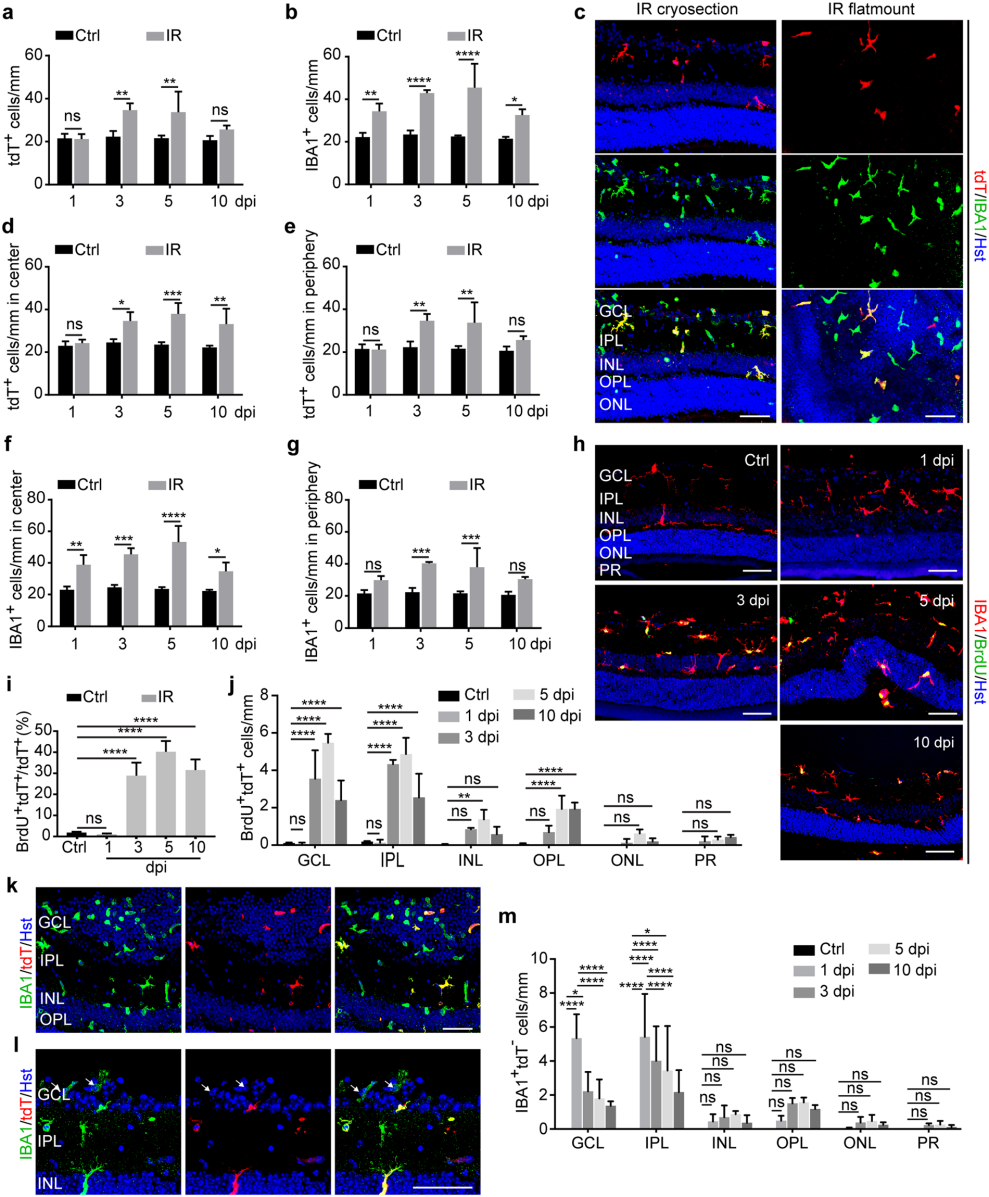
IR induces microglia proliferation and macrophage infiltration. (**a**) IR induces a dynamic increase of tdT^+^ cells. Data were obtained at the indicated time points and presented as mean ± SD per mm retinal length (n = 4 mice per group). Statistical analysis was performed by two-way ANOVA and post hoc Tukey’s test (F(1,21) = 29.04 and p < 0.0001 for injury effect; F(3, 21) = 6.68 and p = 0.0024 for time effect; F(3,21) = 5.31 and p = 0.0070 for time-injury interaction; **p ≤ 0.005; ns, not significant). (**b**) IR induces a dynamic increase of IBA1^+^ microglia/macrophages. Data were obtained at the indicated time points and are presented as mean ± SD per mm retinal length (n = 4 mice per group). Statistical analysis was performed by two-way ANOVA and post hoc Tukey’s test (F(1,21) = 119.7 and p < 0.0001 for injury effect; F(3, 21) = 5.33 and p = 0.0069 for time effect; F(3,21) = 3.62 and p = 0.0300 for time-injury interaction; *p = 0.0270, **p = 0.0066, and ****p < 0.0001). (**c**) Confocal images of microglia/macrophages at 5 dpi. Some IBA1^+^ cells are not co-labeled by tdT. Scale bars, 50 µm. (**d**–**g**) Subregional analysis of increased microglia/macrophages. Data were obtained at the indicated time points and are presented as mean ± SD per mm retinal length (n = 4 mice per group). Statistical analysis was performed by two-way ANOVA and post hoc Tukey’s test (*p ≤ 0.05, **p ≤ 0.01, ***p ≤ 0.001, and ****p ≤ 0.0001; ns, not significant). (**h**) Confocal images of proliferating microglia/macrophages at the indicated time points. Scale bars, 50 µm. (**i**) Quantification of BrdU-labeled microglia. Data were obtained at the indicated time points and are presented as mean ± SD (n = 4 mice per group). Statistical analysis was performed by two-way ANOVA and post hoc Tukey’s test (****p ≤ 0.0001; ns, not significant). (**j**) Retinal layer distribution of proliferating microglia. Data were obtained at the indicated time points and are presented as mean ± SD per mm retinal length (n = 4 mice per group). Statistical analysis was performed by two-way ANOVA and post hoc Tukey’s test (**p = 0.0021 and ****p ≤ 0.0001; ns, not significant). (**k**–**l**) Representative confocal images showing infiltrated macrophages in the GCL at 1 dpi. These cells are IBA1^+^tdT^−^. Scale bar, 50 µm. (**m**) Retinal layer distribution of recruited macrophages after injury. Data are presented as mean ± SD per mm retinal length (n = 4 mice per group). Statistical analysis was performed by two-way ANOVA and post hoc Tukey’s test (*p ≤ 0.05 and ****p ≤ 0.0001; ns, not significant).

Microglia activation was further examined by quantification of cell numbers. Compared to cells in control eyes, the total number of tdT^+^ cells in IR eyes at 3 dpi significantly increased and reached a peak at 5 dpi (Fig. 3a). Similarly, there was a significant increase of IBA1^+^ cells starting at 1 dpi and persisting to 10 dpi (Fig. 3b). Interestingly, the overall number of IBA1^+^ cells was more than that of tdT^+^ cells (Fig. 3a–c). Since nearly all IBA1^+^ cells was initially traced by the tdT reporter under non-injured condition (Fig. 1c), the additional IBA1^+^tdT^−^ cells might originate from IR injury-induced recruitment of circulating monocytes/macrophages. This is consistent with previous reports showing that a major fraction of circulating monocytes/macrophages can lose the inducible reporter at 2 weeks post TAM injection due to their continuous turnover^35,38,39^.

Subregional analysis showed a significant increase of tdT^+^ and IBA1^+^ cells in both the central and peripheral retina after injury, although the increase at 10 dpi was less in the peripheral region (Fig. 3d–g).

IR injury-induced microglia hyperplasia may result from cell proliferation, which was determined by incorporation of 5-bromo-2′-deoxyuridine (BrdU). BrdU was supplied in drinking water starting on the day of IR injury and lasting until the day of analysis. While BrdU^+^ cell were rarely detected in control or IR eyes at 1 dpi (Fig. 3h), around 30% of tdT^+^ cells incorporated BrdU at 3, 5, and 10 dpi (Fig. 3i). Layer distribution analysis showed that tdT^+^ proliferating cells were mainly identified in the GCL and IPL (Fig. 3j).

The monocyte-derived IBA1^+^tdT^−^ macrophages were clearly detectable in the GCL at 1 dpi (Fig. 3k,l). Likewise, layer distribution analysis showed that these cells were mainly found in the GCL and IPL at this time point (Fig. 3m).

### Association of microglia with apoptotic neurons

**Figure 4:**
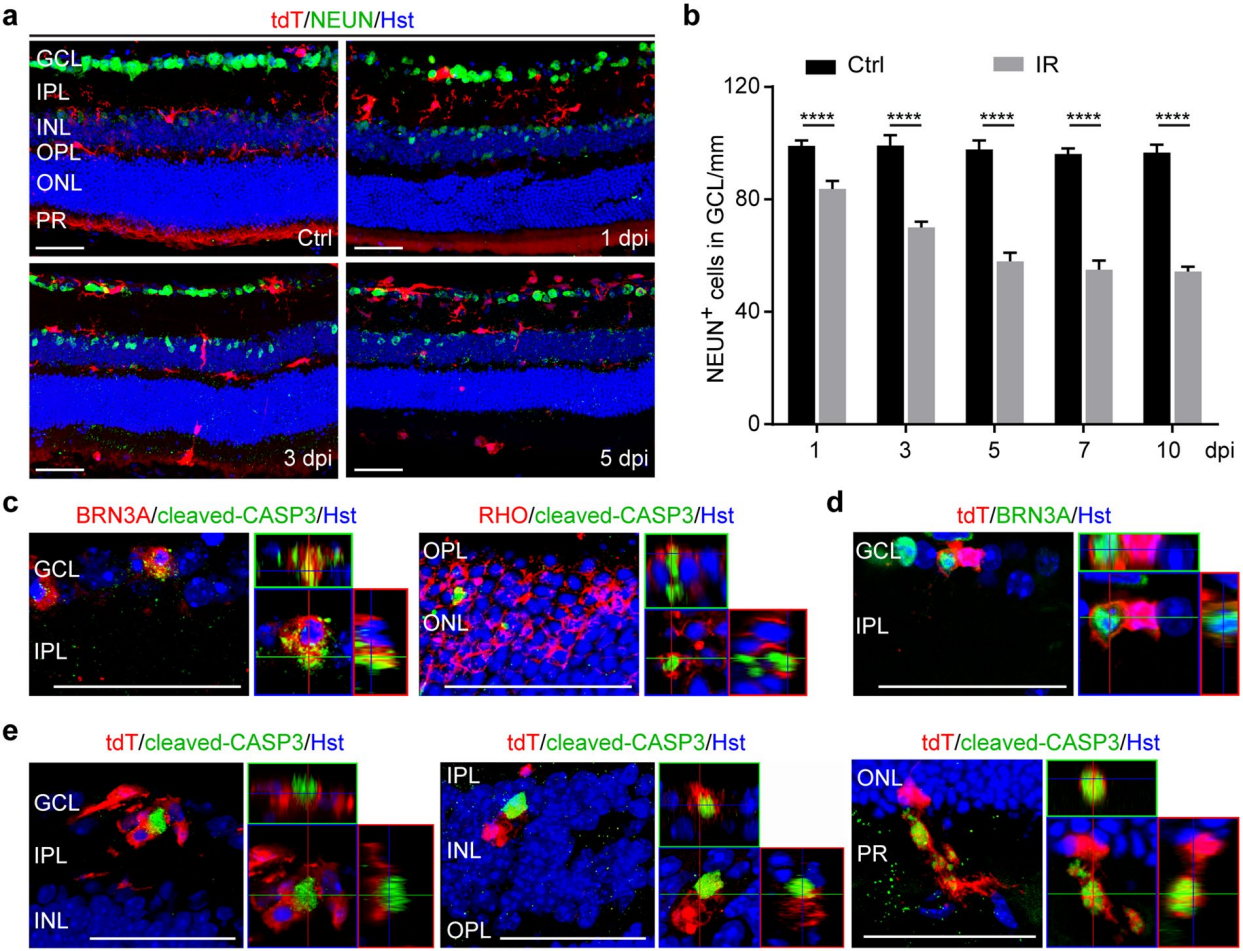
Association of microglia/macrophages with apoptotic neurons. (**a**) Confocal images showing injury-induced progressive loss of neurons in the GCL at the indicated time points. Scale bars, 50 µm. (**b**) Quantification of surviving neurons in the GCL. Data are presented as mean ± SD (n = 4 mice per group). Statistical analysis was performed by two-way ANOVA and post hoc Tukey’s test (F(1,29) = 1,483 and p < 0.0001 for injury effect; F(4, 29) = 51.46 and p < 0.0001 for time effect; F(4,29) = 35.36 and p < 0.0001 for time-injury interaction; ****p < 0.0001). (**c**) Confocal images showing apoptotic neurons at 5 dpi. Scale bars, 50 µm. (**d**) Confocal images showing a retinal ganglion cell engulfed by microglia at 5dpi. Scale bar, 50 µm. (**e**) Tight associations of microglia with apoptotic cells across all retinal layers at 5dpi. Scale bars, 50 µm.

Retinal IR injury causes neuron death mainly in the inner retina, although cells in the outer layers can also be affected^5^. These cells, including retinal ganglion cells (RGCs) and displaced amacrine cells, can be identified by NEUN staining (Fig. 4a). IR injury caused a progressive decrease of NEUN^+^ cells when examined at 1, 3, 5, and 7 dpi (Fig. 4a,b). Two-way ANOVA analysis showed a significant time-dependent loss of neurons in the GCL with injury, although their number stabilized after 7 dpi (Fig. 4b).

Consistently, immunohistochemistry showed that a fraction of BRN3A^+^ RGCs and RHO^+^ rod photoreceptors stained positive for cleaved caspase 3, a marker for cells undergoing apoptosis, when examined at 5 dpi (Fig. 4c). The engulfment of BRN3A^+^ RGCs by tdT^+^ microglia indicates phagocytosis (Fig. 4d). Apoptotic cells were found to be frequently associated with and engulfed by tdT^+^ microglia across all of the retinal layers after injury (Fig. 4e).

### Minocycline protects against retinal degeneration

**Figure 5:**
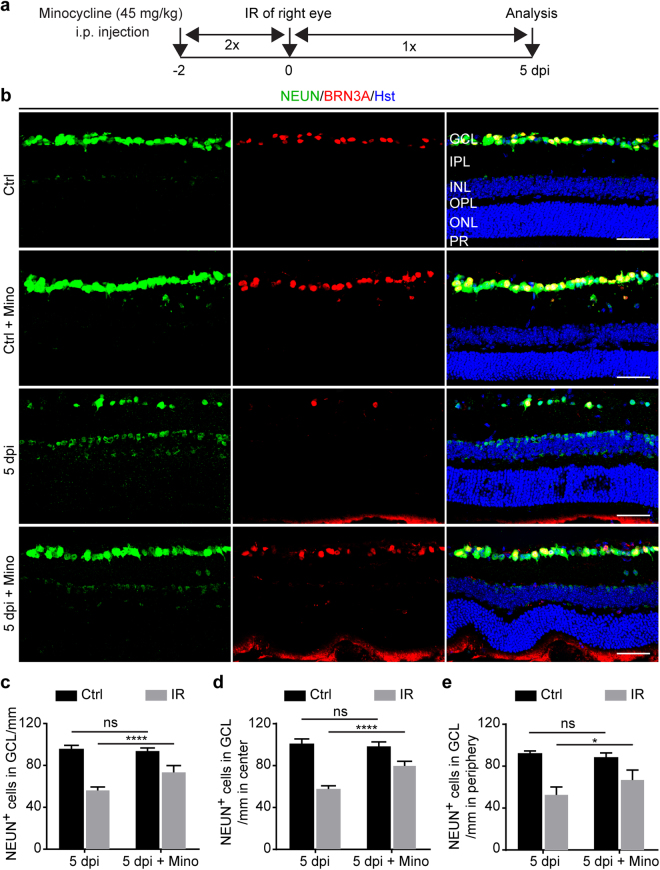
Minocycline improves survival of retinal neurons. (**a**) Experimental design. Minocycline was intraperitoneally (i.p.) injected twice daily for two days prior to IR injury, twice on the day of surgery, and once per day for five days after injury. (**b**) Confocal images showing neurons in the GCL under the indicated treatment conditions at 5dpi. Mino, minocycline. Scale bars, 50 µm. (**c**) Quantification of neurons in the GCL. Data are presented as mean ± SD per mm retinal length (n = 5 mice per group). Statistical analysis was performed by two-way ANOVA and post hoc Tukey’s test (F(1,16) = 246.3 and p < 0.0001 for injury effect; F(1,16) = 15.34 and p = 0.0012 for treatment effect; F(1,16) = 29.47 and p = 0.0001 for injury-treatment interactions; ****p < 0.0001; ns, not significant). (**d**,**e**) Subregional analysis shows a positive effect of minocycline on neuronal survival across the entire retina. Data are presented as mean ± SD per mm retinal length (n = 5 mice per group). Statistical analysis was performed by two-way ANOVA and post hoc Tukey’s test (*p = 0.0152 and ****p < 0.0001; ns, not significant).

We next examined whether retinal degeneration can be ameliorated by altering microglia/macrophages. Minocycline is a well-tolerated antibiotic that can result in attenuation of the activated microglia and accordingly induce neuroprotection in experimental models of several neuronal diseases^25–30^. Two weeks after TAM injections, a cohort of adult *Cx3cr1::CreER* ^*T2*^ *;Ai14* mice were randomly divided into two groups (n = 5/group) with one being treated with minocycline and the other with vehicle control. Two days later, all mice were subjected to IR injury in the right eye while the left uninjured eye served as a control. Minocycline was administered for a total of 8 consecutive days starting at two days prior to IR injury and lasting until 5 dpi (Fig. 5a). Immunohistochemistry was performed to detect BRN3A^+^ RGCs and all NEUN^+^ neurons, which were analyzed by confocal microscopy (Fig. 5b). NEUN^+^ cells in the GCL were specifically quantified to determine the effect of minocycline treatment on overall survival of retinal neurons (Fig. 5c–e).

Two-way ANOVA analysis revealed a significant interaction between injury and treatment groups (Fig. 5C). Post hoc analysis by Tukey’s multiple comparisons test showed that, when compared to vehicle controls, minocycline treatments greatly improved survival of NEUN^+^ cells in the IR injured eyes (p < 0.0001). These surviving neurons could be found throughout the injured retina, both in the central and peripheral regions (Fig. 5d,e).

### Effect of minocycline on microglia/macrophage activation

**Figure 6:**
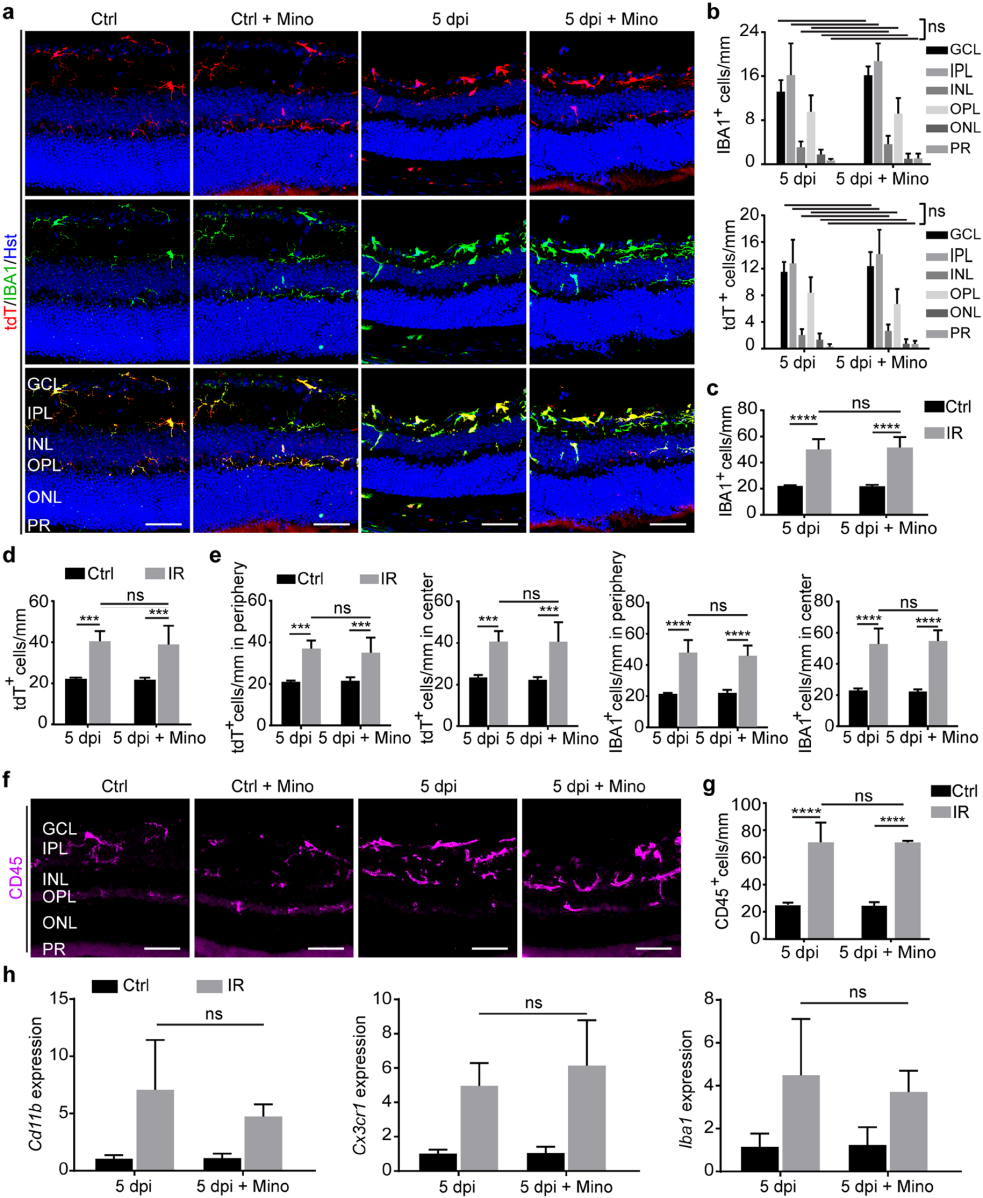
Minimal effect of minocycline on microglia/macrophage activation. (**a**,**b**) Minocycline fails to change morphology and retinal layer distribution of microglia/macrophages at 5 dpi. Data are presented as mean ± SD per mm retinal length (n = 5 mice per group). Statistical analysis was performed by two-way ANOVA and post hoc Tukey’s test (ns, not significant). Scale bars, 50 µm. (**c**,**d**) The overall number of microglia/macrophages is not altered by minocycline after IR. Data are presented as mean ± SD per mm retinal length (n = 5 mice per group). Statistical analysis was performed by two-way ANOVA and post hoc Tukey’s test (***p ≤ 0.0005 and ****p < 0.0001; ns, not significant). (**e**) Minocycline has minimal effect on subregional distribution of activated microglia/macrophages. Data are presented as mean ± SD per mm retinal length (n = 5 mice per group). Statistical analysis was performed by two-way ANOVA and post hoc Tukey’s test (***p ≤ 0.0007, and ****p < 0.0001; ns, not significant). (**f**,**g**) Histological analysis of CD45^+^ microglia/macrophages. Data are presented as mean ± SD per mm retinal length (n = 5 mice per group). Statistical analysis was performed by two-way ANOVA and post hoc Tukey’s test (****p < 0.0001; ns, not significant). (**h**) qRT-PCR analysis of gene expression. Data are presented as mean ± SD (n = 5 mice per group). Statistical analysis was performed by two-way ANOVA and post hoc Tukey’s test (ns, not significant).

We examined the cellular mechanism underlying minocycline-induced neuroprotection after IR injury. Microglia/macrophages were analyzed by immunohistochemistry in *Cx3cr1::CreER* ^*T2*^ *;Ai14* mice after IR injury and treatment with either vehicle or minocycline. In control eyes, IBA1^+^ and tdT^+^ cells exhibited ramified morphology with and without minocycline treatment (Fig. 6a). Microglia morphology noticeably changed after injury as IBA1^+^ and tdT^+^ cells showed soma enlargement with shortening and thickening of their processes. Unexpectedly, minocycline treatment did not produce obvious changes in this overall morphology of the activated IBA1^+^ or tdT^+^ cells (Fig. 6a). Moreover, retinal layer distribution analysis showed insignificant changes in the migration of IBA1^+^ or tdT^+^ cells after injury (Fig. 6b). A quantification of cell numbers also failed to detect major reduction of injury-induced IBA1^+^ or tdT^+^ cells (Fig. 6c,d). A lack of minocycline-induced cellular changes was similarly observed when microglia/macrophages were subdivided into central or peripheral regions (Fig. 6e). These data were further supported with the morphological and quantitative analysis of CD45^+^ microglia/macrophages (Fig. 6f,g).

We also analyzed cell markers by qRT-PCR using a different cohort of mice. Wild-type C57BL/6J mice were randomly divided into two groups (n = 5 per group), each of which underwent treatment and injury as described above (Fig. 5a). Retinas were collected at 5 dpi and processed for qRT-PCR analyses. The expression of *Cd11b*, *Cx3cr1* and *Iba1*, which are well known markers for microglia/macrophages, were greatly induced by IR injury (Fig. 6h). Nonetheless, their expression was not significantly altered by treatment with minocycline (Fig. 6h), consistent with the immunohistochemistry data (Fig. 6a–g).

### Minocycline induces M2 polarization of microglia/macrophages

**Figure 7:**
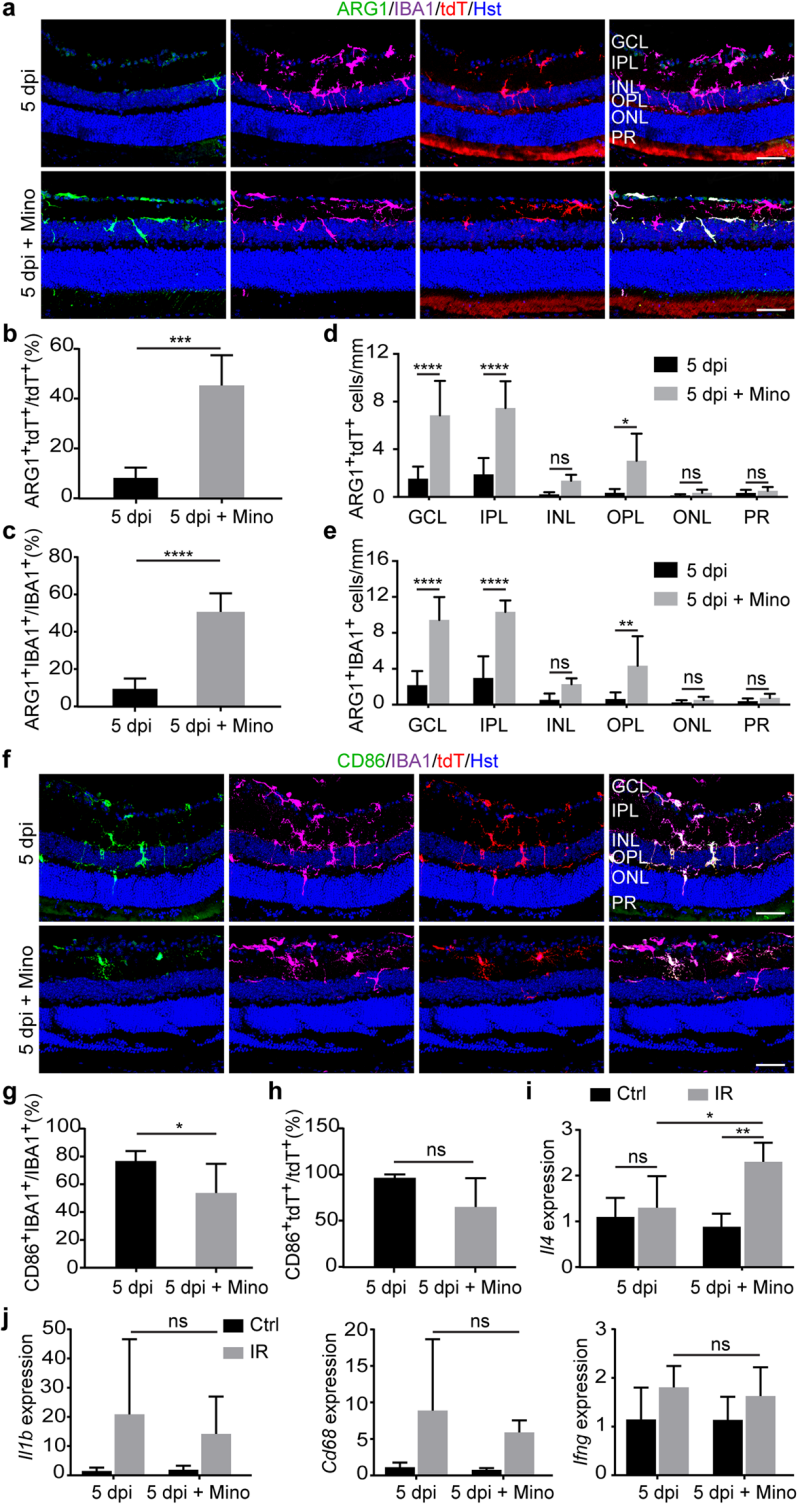
Minocycline affects polarization of microglia/macrophages. (**a**) Confocal images showing ARG1^+^ M2 phenotype of microglia/macrophages at 5 dpi. Scale bars, 50 µm. (**b**,**c**) Minocycline promotes M2 polarization of activated microglia/macrophages. Data are presented as mean ± SD (n = 5 mice per group). Statistical analysis was performed by unpaired two-tailed t-test (***p = 0.0002 and ****p < 0.0001 for ARG1^+^tdT^+^ and ARG1^+^IBA1^+^ cells, respectively). (**d**,**e**) Minocycline-induced M2 phenotype is mainly in the inner retinal layers. Data are presented as mean ± SD per mm retinal length (n = 5 mice per group). Statistical analysis was performed by two-way ANOVA and post hoc Tukey’s test (*p = 0.0173, **p = 0.0027 and ****p < 0.0001; ns, not significant). (**f**) Confocal images showing CD86^+^ M1 phenotype of microglia/macrophages. Scale bars, 50 µm. (**g**,**h**) Minocycline modestly reduces M1 phenotype of microglia/macrophages at 5dpi. Data are presented as mean ± SD (n = 5 mice per group). Statistical analysis was performed by unpaired two-tailed t-test (p = 0.055 and *p = 0.048 for CD86^+^tdT^+^ and CD86^+^IBA1^+^ cells, respectively). (**i**) qRT-PCR analysis of interleukin 4 (*Il4*) expression. Data are presented as mean ± SD (n = 5 mice per group). Statistical analysis was performed by two-way ANOVA and post hoc Tukey’s test (*p = 0.0190 and **p = 0.0012; ns, not significant). (**j**) qRT-PCR analysis of genes for M1 phenotype. Data are presented as mean ± SD (n = 5 mice per group). Statistical analysis was performed by two-way ANOVA and post hoc Tukey’s test (ns, not significant).

To further understand the neuroprotective effect of minocycline after IR injury, we examined microglia/macrophage polarization. The M1 and M2 phenotypes can be specifically identified by staining of CD86 and ARG1, respectively^32,34,40^. A respective 14% and 16% of tdT^+^ and IBA1^+^ cells stained positive for ARG1 in vehicle-treated eyes after IR injury (Fig. 7a–c). In sharp contrast, minocycline treatment significantly increased the percentage of ARG1^+^ cells to 45% and 51% of tdT^+^ and IBA1^+^ cells, respectively (Fig. 7b,c). These ARG1^+^ microglia/macrophages were mainly distributed in the inner layers of the ischemic retina, such as the GCL and IPL (Fig. 7d,e).

Minocycline treatment, on the other hand, decreased the number of CD86^+^ cells (Fig. 7f). Quantification showed a respective 23% and 32% reduction of CD86^+^IBA1^+^ and CD86^+^tdT^+^ cells in IR-injured eyes that were treated with minocycline when compared to vehicle controls (Fig. 7g,h). Nonetheless, the reduction of CD86^+^tdT^+^ cells was less significant (p = 0.055).

Genes involved in microglia/macrophage phenotypes were also analyzed by qRT-PCR using retinas under several conditions. *Il4*, a well-established cytokine capable of stimulating M2 polarization^41–43^,was significantly induced by minocycline under IR injury condition (Fig. 7i). In contrast, minocycline had minimal effect on the expression of *Il1b*, *Cd68*, or *Ifng* (Fig. 7j). Together, these data indicate that reactive microglia/macrophages were shifted toward an M2 phenotype by minocycline after IR retinopathy.

### IL4 is neuroprotective

**Figure 8:**
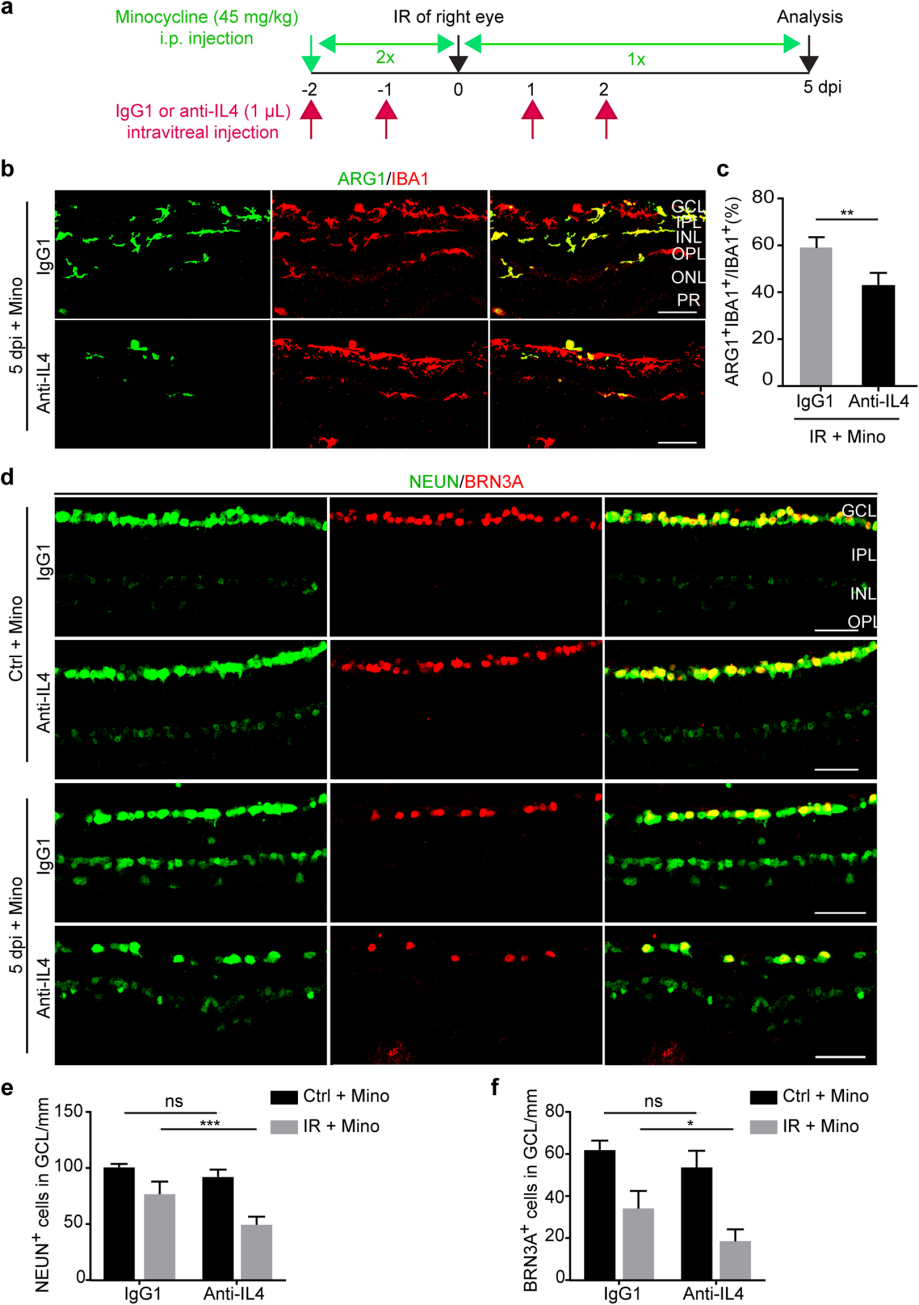
A role of IL4 in microglia/macrophage polarization and neuron survival. (**a**) Experimental design. (**b**,**c**) IL4 neutralization dampens minocycline-induced microglia/macrophage polarization. Immunohistochemistry was performed at 5 dpi. Data are presented as mean ± SD (n = 5 mice per group). Statistical analysis was performed by unpaired two-tailed t-test (**p = 0.0023). Scale bars, 50 µm. (**d**–**f**) IL4 neutralization reduces minocycline-mediated neuroprotection. Data are presented as mean ± SD per mm retinal length (n = 5 mice per group). Statistical analysis was performed by two-way ANOVA and post hoc Tukey’s test (*p = 0.0117, and ***p = 0.0001; ns, not significant).

The role of minocycline-induced IL4 was examined by using an IL4-neutralizing monoclonal antibody^44,45^. Wild-type C57BL/6J mice were randomly divided into two groups (n = 5 per group) and subjected to IR injury in the right eye while the left uninjured eye served as a control. All mice were treated with minocycline from two days prior to IR injury until analysis (a total of 8 days). These two groups were also intravitreally injected in both eyes with either the IL4-neutralizing antibody or the IgG1 control (Fig. 8a). At 5 dpi, these eyes were analyzed by immunohistochemistry and confocal microscopy. When compared to the IgG1 controls, IL4-neutralization resulted in a significant decrease of ARG1^+^ microglia/macrophages in minocycline-treated IR eyes (Fig. 8b,c). Very interestingly, blocking IL4 function also markedly reduced the number of NEUN^+^ or BRN3A^+^ retinal neurons in these eyes (p = 0.0001 and p = 0.0117, respectively; Fig. 8d–f). Together, these results indicate that IL4 is neuroprotective and may play a role in minocycline-induced microglia/macrophage polarization and neuronal survival after IR.

## Discussion

Neurological disorders can activate microglia, which, together with recruited macrophages, exert either beneficial or detrimental effects^20,29,31,46–50^. Activation of microglia/macrophages was also analyzed in several experimental models of retinal degeneration such as retinitis pigmentosa, light-induced retinopathy, and experimental glaucoma^37,51,52^. Nevertheless, it is less studied in the retinal IR injury model^53–55^. The behavior of retinal microglia/macrophages and their role in disease progression after IR remain unresolved. Our systematic analysis of the cellular response reveals that IR induces profound transient activation of resident microglia and the recruitment of circulating macrophages. These cells associate with apoptotic neurons after IR. Most importantly, our results demonstrate that minocycline is neuroprotective and can polarize microglia/macrophages toward an M2 phenotype. Such beneficial effect of minocycline seems to require IL4 function.

The *Cx3cr1::CreER* ^*T2*^ *;Ai14* mouse line can be used to distinguish resident microglia from circulating monocyte-derived cells^35,38,39^. A transient TAM treatment leads to Cre-dependent expression of the reporter tdT, which labels both monocytes and microglia. In contrast to resident microglia, which can self-renew and are long-lived, tdT^+^ monocytes are short-lived due to their continuous turnover in circulation^46^. As such, only about 10–15% monocytes/macrophages could be traced 2 weeks after TAM administration^35^. A time course analysis showed that a significant number of IBA1^+^tdT^−^ monocytes was initially found near the inner retina after IR. The total number of recruited monocytes might be underestimated, since some of them may still be traced with tdT at 2 weeks after the last injection of TAM. Such leukocyte recruitment was also reported in IR^56^ and light-induced retinopathy^52^. Our BrdU-dependent pulse-labeling experiments further revealed that around 30% of local tdT^+^ cells underwent proliferation at 3dpi and later. Together, these results indicate that both leukocyte recruitment and local microglia proliferation contribute to IR-induced transient increase of retinal microglia/macrophages.

Phagocytosis is a key function of activated microglia/macrophages. We observed that these cells are frequently associated with and engulf apoptotic neurons across multiple retinal layers after IR injury. Clearing the dead and dying cells through phagocytosis is believed to be a beneficial process^19^. Nevertheless, emerging evidence suggests that phagocytosis may be a double-edged sword. Recent studies show that inflammatory stress exposes viable neurons to phosphatidylserine with resultant phagocytosis of stressed-but-viable neurons^18^. Prevention of phagoptosis by blocking the phagocytic signaling is beneficial for cerebral ischemia^19^. A tight association with dying neurons is consistent with a detrimental effect exerted by activated microglia/macrophages under severe neural injury such as ischemic stroke^57,58^, spinal cord injury^59^, and optic nerve injury^60^.

Our results show that minocycline, an antibiotic with a known inhibitory effect on microglia activation^27–31,61,62^, is neuroprotective in our injury model, consistent with other IR studies^63–66^. The beneficial effect of minocycline is also reported in models of light-induced retinal degeneration^26^, glaucoma^67,68^, diabetic retinopathy^25^ and an ischemic model of branch retinal vein occlusion^69^. In direct contrast to our results, however, Abcouwer *et al*. showed that minocycline inhibited cellular inflammation while it had no significant effect on neurodegeneration in a rat IR model^56^. Their results were unexpected owing to previously reported neuroprotective effect of minocycline on RGC survival in other ischemic models^70^. Differences on IR injury severity and the duration of drug administration may account for the observed discrepancy on the effect of minocycline. Abcouwer *et al*. employed a 45-minute ischemic insult on their rat model, whereas the ischemia duration in our mouse model was 60 minutes. It is well known that extended ischemia causes more severe RGC damage^71^. The neuroprotective effect of minocycline may be more revealing under such a severe damage condition. Moreover, we continually applied minocycline for 5 additional days after IR based on our observation that the increase of microglia/macrophages reaches a peak at 5 dpi. In contrast, Abcouwer *et al*. only applied minocycline for 2 additional days post injury. For potential clinical applications, future detailed comparisons on injury types and drug regimens are clearly needed.

It is somewhat unexpected that minocycline fails to inhibit microglia activation in our IR model. This result is, nonetheless, consistent with what has been previously reported in several other injury models^72–76^. Differences in disease models and drug dosage may account for the observed discrepancy on the effectiveness of minocycline on microglia activation^74,75^. In contrast, our results reveal that minocycline induces polarization of microglia/macrophages toward an M2 phenotype in our IR model. Imbalance of the M1 and M2 phenotype (especially a lack of proper M2 phenotype) is associated with neurodegeneration including ischemic stroke, traumatic brain injury, spinal cord injury, and Alzheimer’s disease^24,77^. Human eyes with age-related macular degeneration have increased M1/M2 ratios when compared to age-matched healthy controls^78^. Conversely, enhanced M2 polarization is generally neuroprotective in several other neuropathy models^77,79,80^.

IL4 seems to be a critical mediator of minocycline’s beneficial effect in our IR model. Consistent with a role of IL4 in stimulating macrophage M2 polarization^41–43^, we found that IL4 neutralization decreased minocycline-induced M2 phagocytes and neuronal survival after IR. Nonetheless, it should be noted that minocycline may exert neuroprotection through multiple mechanisms, such as inhibition of caspase-1, caspase-3, matrix metalloproteinases, p38 mitogen-activated protein kinase, and inducible form of nitric oxide synthase (iNOS)^81^.

Together, our results reveal dynamic behaviors of genetically traced resident microglia and acutely recruited macrophages in a mouse retinal IR model. Importantly, minocycline treatment induces M2 polarization of microglia/macrophages and mitigates neuron loss after IR. Such beneficial effect of minocycline seems to be at least partially mediated by IL4 function. Given a well-established clinical safety profile, minocycline has a therapeutic potential for treating retinal degenerative diseases. Future studies are clearly warranted to better understand the underlying molecular mechanisms.

## Materials and Methods

### Animals

Wild-type male C57BL/6J mice were purchased from The Jackson Laboratory. *Cx3cr1:CreER* ^*T2*^ ^82^ and *Ai14* (Rosa-tdTomato)^36^ transgenic mice were previously described and obtained from The Jackson Laboratory (Bar Harbor, ME). Adult male and female mice at 2–3 months of age were used unless otherwise stated. All mice were housed in the UT Southwestern animal facility with a 12 h light/dark cycle and ad libitum access to food and water. All experimental protocols and procedures were approved by the Institutional Animal Care and Use Committee at UT Southwestern. All methods were performed in accordance with the relevant guidelines and regulations.

### Tamoxifen and BrdU Administration

Tamoxifen (T5648; Sigma-Aldrich, St. Louis, MO) was dissolved in a mixture of ethanol and sesame oil (10:90 by volume) at a concentration of 40 mg/ml and stored at 4°C. It was intraperitoneally injected at a daily dose of 0.1 mg/g body weight for 4 days. Proliferating cells were labeled by 5-bromo-2′-deoxyuridine (BrdU; H27260; Alfa Aesar, Ward Hill, MA) in drinking water (0.5 g/l) for the indicated durations.

### Retinal Ischemia-Reperfusion

Retinal ischemia-reperfusion (IR) injury was induced as previously described^83^. Briefly, mice were randomly selected and anesthetized with an intraperitoneal (i.p.) injection of a cocktail of ketamine (120 mg/kg) and xylazine (116 mg/kg). A 1% tropicamide saline solution (Mydriacil; Alconox, New York, NY) and 0.5% proparacaine hydrochloride (Alcaine; Alcon Inc., Fort Worth, TX) eye drops were topically administered to dilate and anesthetize the right eyes, respectively. A 32-gauge needle attached to a sterile saline-filled reservoir was inserted into the anterior chamber through the cornea of the right eye. Pressure in the eye was increased to 80–90 mm Hg with a pressure infuser (Infu-surg; Ethox Corp., Buffalo, NY) for 1 hour to induce retinal ischemia. The successful achievement of retinal ischemia was confirmed by whitening of the iris and loss of red reflex. After ischemia, the needle was removed to allow the IOP to normalize and the eye to reperfuse with visual confirmation of the reflow of retinal circulation. Contralateral left eyes served as controls. Mice were placed on a heating pad for the duration of the procedure and recovery. During recovery from anesthesia, the mice were placed in their home cages and B.N.P. Triple Antibiotic (Bacitracin, Neomycin, Polymyxin B; Akorn Animal Health, Inc., Lake Forest, IL) and artificial tear Ophthalmic Ointment were applied to the cornea to prevent corneal desiccation and infection. Mice without immediate reperfusion after the ischemic period or those with lens injuries or cataracts were excluded from the study.

### Systemic Minocycline Treatment

A minocycline treatment regimen was employed as previously described in several studies^84^. Minocycline (M9511; Sigma-Aldrich) was administered by intraperitoneal (IP) injections, with three initial twice-daily dosages (45 mg/kg) starting two days prior to ischemia and once daily for 5 days after ischemia.

### IL4 Neutralization

Antibodies were administered through intravitreal injections with a procedure as previously described^85^. Affinity purified rat anti-mouse IL4 monoclonal antibody (clone 11B11; IgG1, κ Isotype; 1.0 mg/ml; Cat#504108; Biolegend, San Diego, CA) and a similarly purified control rat monoclonal antibody (clone RTK2071; IgG1, κ Isotype, 1.0 mg/ml; Cat#400414; Biolegend, San Diego, CA) were used. One µl antibody was injected into each eye of minocycline treated C57BL/6J mice. The injections were conducted once daily for two days both prior to and after ischemia (a total of 4 injections for each eye).

### Immunohistochemistry

Mice were euthanized by CO_2_ overdose and intracardially perfused first with phosphate-buffered saline (PBS) and then 4% paraformaldehyde (PFA) in PBS. Both ischemic and contralateral control eyes of each animal were gently enucleated. For whole mount, retinas were isolated and fixed for 1 hour in 4% PFA at room temperature (RT). For sections, eyes were post-fixed overnight and cryoprotected with 30% sucrose at 4 °C for 24–48 hours. Cryosections at 20 ųm thickness were placed on Superfrost Plus slides (Thermo Fisher Scientific, Houston, TX) and allowed to dry at RT for 10 min. After washing the isolated retinas or the sections 3 times in PBS, they were blocked with blocking solution (3% BSA and 0.2% Triton X-100 in PBS) for 1 h at RT. Primary antibodies were applied on them for at least 24 h at 4 °C followed by three rinses with PBST buffer (0.2% Triton X-100 in PBS). The following primary antibodies were used: IBA1 (019-19741; rabbit; 1:200; Wako), BrdU (OBT0030; rat BU1/75; 1:300; Accurate Chemical), rhodopsin (RHO; MAB5356; mouse; 1:200; Chemicon), NEUN (ab177487; rabbit; 1:1000; Abcam), BRN3A (MAB1585; mouse; 1:200; Millipore), cleaved-caspase3 (cleaved-CASP3; 9661; rabbit; 1:200; Cell Signaling), arginase I (ARG1; sc-18354; goat; 1:100; Santa Cruz), CD86 (550542; rat; 1:100; BD Pharmingen), CD45(550539; rat; 1:200; BD Pharmingen). Tissues were subsequently incubated with Alexa Fluor 594-, 488-, or 647-conjugated secondary antibodies (1:500; from Jackson ImmunoResearch, West Grove, PA) for 3 h at RT. Nuclei were counterstained with Hoechst 33342 (Hst). After triple washing with PBST buffer, the samples were mounted and cover-slipped with 2.5% PVA-DAPCO anti-fading medium and examined by a Zeiss LSM700 confocal microscope. Representative data are shown from at least three similar images.

### Cell Counting

NEUN^+^ cells were quantified from 6 micrograph images (three continuous images from each side of the optic nerve head) with about 340 mm retinal length per area as previously described^86^. IBA1^+^, tdT^+^, BrdU^+^, ARG1^+^, CD86^+^ or CD45^+^ cells were imaged along the full length of retinal cryosections cut in the vertical plane (naso-temporal) including both central and peripheral areas of the retina. Marker-positive cell bodies were evaluated on each retinal section with the consideration of their distribution characteristics within the retinal layers. Confocal Z-stack of each image collected over a depth of 20 μm was projected as one composite image. A Cell Counter software plugin in the ImageJ program was used for cell counting. The total number of marker-positive cells for each retina was the average of all analyzed sections obtained at comparable locations from both the superior and inferior retina. The results are reported as number of cells per mm retinal length. All of the data were obtained from 3–5 mice in each group.

### qRT-PCR

Total retinal RNAs were homogenized and isolated using TRIzol Reagent (Invitrogen) and an RNA extraction kit (Zymo Research, Irvine, CA). cDNA synthesis was generated using the SuperScript® III RT First Strand Kit (Invitrogen, Carlsbad, CA). RT–PCR using independent RNA samples in duplex was performed using SYBR Green chemistry (Invitrogen). The primer sequences are listed in Supplementary Table S1. The expression of *Hprt* was used as an internal control for normalization. The relative difference in target gene expression was calculated using the following formula: ΔΔCT = ΔCT (target gene) - ΔCT (*Hprt*) and then normalized to the control left eye.

### Statistical Analysis

Statistical analysis was performed with the GraphPad Prism software (GraphPad Inc., La Jolla, CA). Statistical differences among four or more groups, such as comparing cell number at different time points for experimental groups, were analyzed by two-way ANOVA and Tukey’s multiple comparisons test. The effects of minocycline treatment on ischemic groups were analyzed by unpaired two-tailed t-test. The data were expressed as mean ± SD. Differences were considered statistically significant at p value ≤ 0.05.

## Electronic supplementary material

Supplementary Table S1
