# Supplementary material for: Minocycline modulates microglia polarization in ischemia-reperfusion model of retinal degeneration and induces neuroprotection
Source: Sci Rep. 2017 Oct 25;7:14065. doi: 10.1038/s41598-017-14450-5 (PMC5656679; doi:10.1038/s41598-017-14450-5)
Supplement: Supplementary file 1 — Supplementary Table S1 [file 41598_2017_14450_MOESM1_ESM.pdf]

Minocycline modulates microglia polarization in ischemia-reperfusion model of retinal degeneration and induces neuroprotection

Amel Ahmed[1,3], Lei-Lei Wang[1,2], Safaa Abdelmaksoud[3], Amal Aboelgheit[3], Safaa Saeed[3], and Chun-Li Zhang[1,2*]

[1]Department of Molecular Biology, University of Texas Southwestern Medical Center, Dallas, Texas 75390, USA

[2]Hamon Center for Regenerative Science and Medicine, University of Texas Southwestern Medical Center, Dallas, Texas 75390, USA

[3]Department of Histology and Cell Biology, Faculty of Medicine, Assiut University, Assiut, Egypt

*Corresponding author

Chun-Li Zhang, Ph.D.

Tel: (214)-648-1670

Fax: (214)-648-1488

Email: Chun-Li.Zhang@UTSouthwestern.edu

Supplementary Table S1. Primer sequences for qPCR

Genes	Sequences
Cd11b	forward primer, 5′- AAGGATTCAGCAAGCCAGAA-3′
	reverse primer, 5′-GGAGGGATGAGAG TCCACAT-3′
Cx3cr1	forward primer, 5′-CAGCATCGACCGGTACCTT-3′
	reverse primer, 5′-GCTGCACTGTCCGGTTGTT-3′
Iba1	forward primer, 5′-CTTGAA GCGAATGCTGGAGAA-3′
	reverse primer, 5′-GGAGCCACTGGACACCTCTCT-3′
Il4	forward primer 5′-TGGGTCTCAACCCCCAGCTAGT-3′
	reverse primer 5′-TGCATGGCGTCCCTTCTCCTGT-3′
Cd86	forward primer 5′-ACGATGGACCCCAGATGCACCA-3′
	reverse primer 5′- GCGTCTCCACGGAAACAGCA-3′
Il1b	forward primer 5′-CCTGCAGCTGGAGAGTGTGGAT-3′
	reverse primer 5′- TGTGCTCTGCTTGTGAGGTGCT-3′
Cd68	forward primer, 5′-CCACAGGCAGCACAGTGGACA-3′
	reverse primer 5′-TCCACAGCAGAAGCTTTGGCCC-3′
Infg	forward primer, 5′- GCCAAGTTTGAGGTCAACAACCCA-3′
	reverse primer 5′- CCCACCCCGAATCAGCAGCG-3′
